# High-resolution structures of the SARS-CoV-2 2’-O-methyltransferase reveal strategies for structure-based inhibitor design

**DOI:** 10.1126/scisignal.abe1202

**Published:** 2020-09-29

**Authors:** Monica Rosas-Lemus, George Minasov, Ludmilla Shuvalova, Nicole Inniss, Olga Kiryukhina, Joseph Brunzelle, Karla J. F. Satchell

**Affiliations:** 1 Department of Microbiology-Immunology, Northwestern University, Feinberg School of Medicine, Chicago, IL, USA; 2 Center for Structural Genomics of Infectious Diseases, Northwestern University, Feinberg School of Medicine, Chicago, IL, USA; 3 Northwestern Synchrotron Research Center, Life Sciences Collaborative Access Team, Northwestern University, Argonne, IL 60439, USA.

## Abstract

There are currently no antiviral therapies specific for SARS-CoV-2, the virus responsible for the global pandemic disease COVID-19. To facilitate structure-based drug design, we conducted an X-ray crystallographic study of the SARS-CoV-2 nsp16-nsp10 2′-*O*-methyltransferase complex, which methylates Cap-0 viral mRNAs to improve viral protein translation and to avoid host immune detection. We determined the structures for nsp16-nsp10 heterodimers bound to the methyl donor *S*-adenosylmethionine (SAM), the reaction product *S*-adenosylhomocysteine (SAH), or the SAH analog sinefungin (SFG). We also solved structures for nsp16-nsp10 in complex with the methylated Cap-0 analog m^7^GpppA and either SAM or SAH. Comparative analyses between these structures and published structures for nsp16 from other betacoronaviruses revealed flexible loops in open and closed conformations at the m^7^GpppA-binding pocket. Bound sulfates in several of the structures suggested the location of the ribonucleic acid backbone phosphates in the ribonucleotide-binding groove. Additional nucleotide-binding sites were found on the face of the protein opposite the active site. These various sites and the conserved dimer interface could be exploited for the development of antiviral inhibitors.

## Introduction

On December 31^st^, 2019, the World Health Organization (WHO) was alerted of a pneumonia outbreak with an unknown etiology, originating in the Chinese province of Wuhan, Hubei. The etiological agent was identified as a coronavirus, closely related to the virus responsible for Severe Acute Respiratory Syndrome (SARS). The new SARS coronavirus-2 (SARS-CoV-2) causes the severe respiratory infection, Coronavirus Disease 2019 (COVID-19). Within four months, SARS-CoV-2 rapidly spread, sparking a global pandemic. The COVID-19 pandemic has also forced governments to enact “stay-at-home” orders around the world, seriously damaging the global economy (1). According to the World Health Organization, nearly 25 million SARS-CoV-2 infections have been confirmed, of which more than 800,000 were fatal as of Aug 30, 2020 (www.who.int). These data are similar to those from the Johns Hopkins University tracking system (2).

Members of the coronaviridae family of viruses infect birds and mammals, including bats, camels, pigs, and humans. In humans, pathogenic coronaviruses cause acute and severe gastrointestinal infections, fevers, and organ failure. Three of the seven human-tropic coronaviruses – hCoV-229E, hCoV-NL63, and hCoVB-OC43 – cause only asymptomatic or mild infections, including the common cold (3). Four other human coronaviruses are linked to severe infections, including hCoV-HKU1, a common cause of pneumonia; SARS-CoV, with a 10% mortality rate; Middle East Respiratory Syndrome Virus (MERS-CoV); with a 37% mortality rate; and SARS-CoV-2, currently with ~3% mortality rate among confirmed cases (3, 4). Among them, SARS-CoV-2 stands as the one with highest transmissibility, making its containment very difficult (5). As SARS-CoV-2 continues to spread, the need for effective vaccines and therapeutics increases. Therefore, it is urgent to study SARS-CoV-2 mechanisms of infection and replication in order to find effective targets for drug and vaccine development.

Coronaviruses have a large (~30 kb) single-stranded, positive RNA genome that is 5′-capped, and contains a 3′-poly-A tail. The *orf1a* and *orf1ab* coding regions are directly translated, whereas the rest of the genome serves as template to generate sub-genomic messenger RNAs (mRNAs) transcribed from the 3’-end, which are later capped and translated (6–8). The first open reading frame (*orf1a*) produces the large non-structural polyprotein 1a (pp1a), and a programmed -1 ribosomal frameshift results in translation of the larger non-structural polyprotein 1ab (pp1ab) from the reading frame *orf1ab*. These polyproteins are subsequently processed into sixteen non-structural proteins (nsp1–nsp16) that assemble to form the replication-transcription complex (RTC) or function as accessory proteins necessary for viral replication (8, 9).

The components of the RTC include enzymes that regulate mRNA and genomic RNA synthesis, proofreading, and mRNA maturation. Two of these enzymes, nsp14 and nsp16, are critical for capping viral mRNAs, a tactic employed by multiple RNA viruses to avoid immune detection (10). In eukaryotic cells, mRNA capping is initiated by an RNA triphosphatase (TPase), which removes the γ-phosphate from the 5′-end of the nascent mRNA transcript, generating a diphosphate 5′-ppN end. An RNA guanylyltransferase (GTase) subsequently catalyzes the hydrolysis of pyrophosphate (PPi) from a guanidine triphosphate (GTP) molecule, thus forming guanidine monophosphate (GMP). This is followed by the transfer of the α-phosphate of GMP to the diphosphate 5′-ppN transcript end, thus forming the cap core structure, methylguanine-triphosphate-ribonucleotide, referred to as GpppN. GpppN formation is followed by N^7^-methylation of the capping guanylate by a guanine-N^7^-methyltransferase (N^7^-MTase) to irreversibly generate the methylated Cap-0. Further methylation at the ribose 2′-*O* position of the first nucleotide of the RNA is catalyzed by a ribose 2′-*O*-methyltransferase (2′-*O*-MTase) to generate Cap-1 and sometimes at the second nucleotide to generate Cap-2. Both the N^7^-MTase and 2′-*O*-MTase use S-adenosyl-L-methionine (SAM) as the methyl group donor (4, 10).

For coronavirus mRNA maturation, the TPase activity is mediated by nsp13 (6, 11–13), and a still-elusive GTase guanylates the 5’-end of the nascent mRNA. The viral non-structural protein 14 (nsp14), which has N^7^-MTase activity, then generates the Cap-0 (14). Nsp14 is a bifunctional enzyme with independent N^7^-MTase and exonuclease domains (15). The association of nsp14 with viral non-structural protein 10 (nsp10) specifically stimulates nsp14 exonuclease activity but has no effect on the N^7^-MTase activity (16). The coronavirus mRNAs are further modified to have a Cap-1 by the viral 2′-*O*-methyltransferase (nsp16). Nsp16 is a 7-methylguanine-triphosphate-adenosine (m^7^GpppA)-specific, SAM-dependent 2′-*O*-MTase (17, 18) that is activated upon binding to nsp10 (16, 19). Nsp10 is a stable monomeric protein that can also form dodecamers (20, 21) in addition to binding to nsp14 and nsp16 (16, 22). Although no specific enzymatic activity has been identified for nsp10, it is known that nsp10 is a zinc-binding protein and can bind RNA (20, 23, 24). It has also been found that nsp10 interacts with human adaptor protein complex 2 when expressed in mammalian cells (25). However, the main known function of nsp10 is the stabilization of the SAM binding pockets of nsp16 and nsp14 (19). The 2’-*O*-methylation of coronavirus RNA that is mediated by the nsp16-nsp10 heterodimer is essential for preventing recognition by the host to evade immune responses that are triggered by viral mRNAs (17).

Structures of the nsp16-nsp10 complex have been determined for SARS-CoV and MERS-CoV (18, 23, 26, 27), and the analyses elucidated the structural basis for substrate binding and the proposed S_N_2-mechanism of methyl transfer. In order to facilitate structure-based inhibitor design, we initiated a project to determine the structures of the 2′-*O*-MTase from SARS-CoV-2 in complex with its ligands. Herein we present a comprehensive X-ray crystallographic study of the structure of the SARS-CoV-2 nsp16-nsp10 heterodimer. The structures of the heterodimer were determined in complex with the methyl donor SAM, the product of the reaction (S-adenosylhomocysteine, SAH), and pan-methyltransferase inhibitor sinefungin (SFG). In addition, we describe the first publicly deposited SARS structures of nsp16-nsp10 in complex with the mRNA cap m^7^GpppA, which facilitates detailed analysis of the changes in the conformation of flexible loops of nsp16 upon substrate binding. Furthermore, we report crystal structures with sulfate ions in the proposed RNA binding groove as well as several additional nucleotide and sugar binding sites outside the active site. Because nsp16 is one of the most conserved proteins of SARS-CoV-2 and related viruses, these high-resolution structures are expected to be useful as models for developing new antiviral therapeutics to treat COVID-19 and other diseases caused by coronaviruses.

## Results

### High-resolution structures of the SARS-CoV-2 2’-O-MTase heterodimer in two crystal forms

**Fig. 1.**
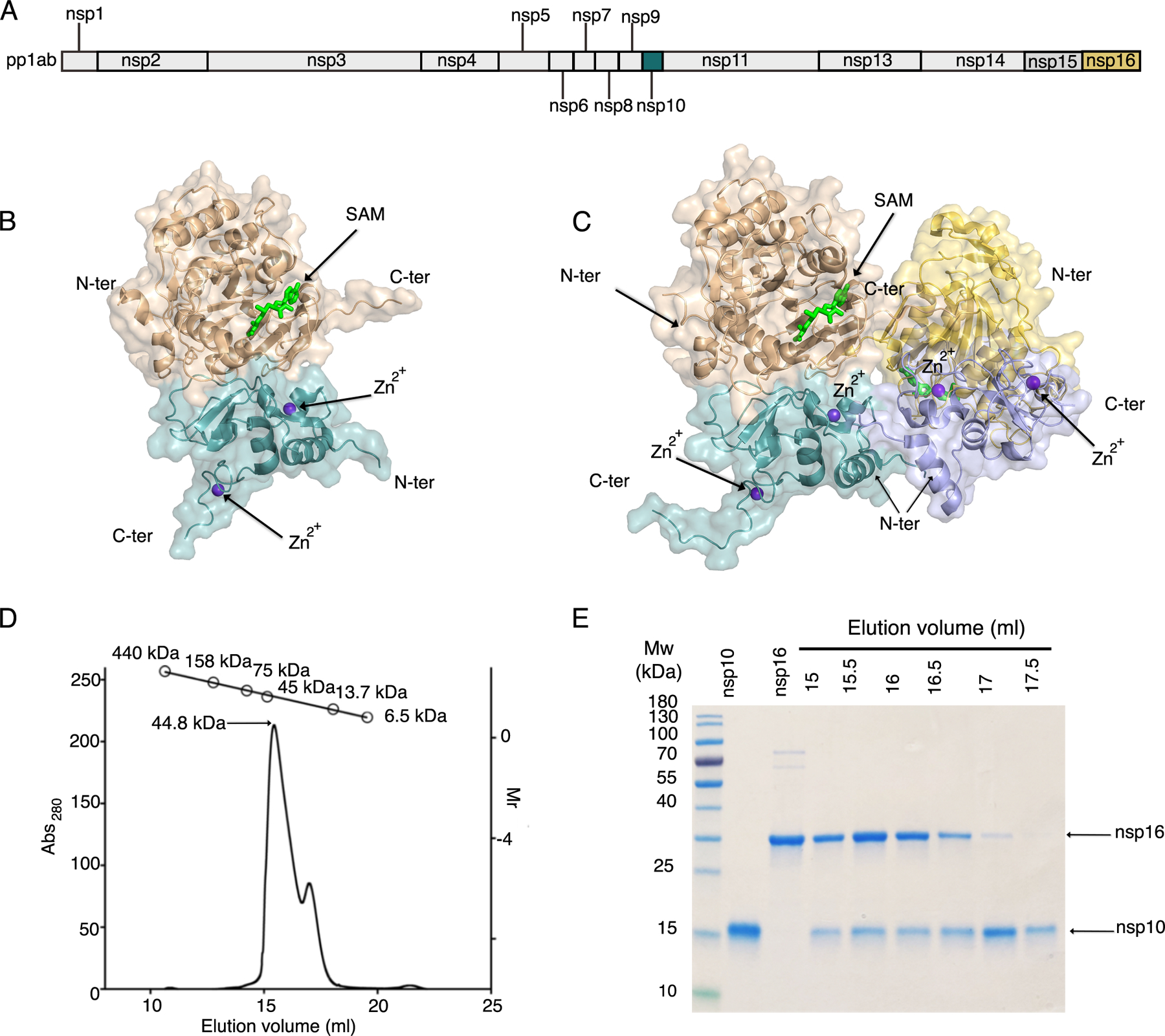
Overall structure of the nsp16-nsp10 oligomers. **(A)** Linear schematic of the *orf1a/orf1b* protein product pp1ab prior to proteolytic processing. **(B)** Cartoon representation of the nsp16-nsp10 heterodimer of the small unit cell crystal form (PDB code 6W4H). **(C)** Cartoon representation of the two nsp16-nsp10 heterodimers in the asymmetric unit of the large unit cell crystal form (PDB code 6W75). In (B) and (C), nsp16 is in shades of tan and yellow and nsp10 is in shades of teal and purple. Ligands are represented as sticks, with SAM in bright green and Zn^2+^ in purple. N-ter, N terminus; C-ter, C terminus. **(D)** Elution profile for analytical size-exclusion chromatography (SEC) with corresponding plot for molecular weight standards shown at the top. **(E)** Separation of elution fractions on a 4–15% gradient SDS-PAGE gel stained with Coomassie blue.

The SARS-CoV-2 proteins nsp10 and nsp16 are encoded by the polycistronic *orf1ab* of the (+) ssRNA (Fig. 1A) and are released from the polyproteins pp1a and pp1ab by the protease nsp5 (28). The protein nsp10 is 14.8 kDa protein released from both polyproteins pp1a and pp1ab, whereas nsp16 is a 33.3 kDa protein released only from the pp1ab polyprotein, which is created by a -1 ribosome shift (28) (Fig. 1A). We successfully expressed and purified recombinant nsp16 and nsp10 separately, combined them 1:1 in the presence of SAM to form nsp16-nsp10 complexes that were set up for crystallization, and obtained diffraction-quality crystals under several conditions (Table S1).

The first structure of the nsp16-nsp10 complex from SARS-CoV-2 was solved at 1.8 Å [RCSB Protein Data Bank (PDB) code 6W4H, Table S1, and Fig. 1B]. The crystal belonged to the space group P3_1_21 with two polypeptide chains in the asymmetric unit, with chain A (nsp16) and chain B (nsp10) forming a heterodimer. We refer to this crystal form as the “small unit cell”. The heterodimer had a total solvent-exposed surface area of 19,710 Å^2^ and a buried area of 2870 Å^2^ estimated by the Protein, Interfaces, Structures and Assemblies tool (PISA), and it is mainly stabilized by hydrophobic interactions and hydrogen bonds at the interface of nsp16 and nsp10. In this structure, the methyl donor SAM was bound to nsp16, and Zn^2+^ was bound to nsp10 (Fig. 1B).

The second crystal form of the nsp16-nsp10 complex with SAM yielded a structure solved at 1.95 Å (PDB code 6W75, Table S2). This crystal form belonged to the P3_2_21 space group and had four chains in the asymmetric unit. The four chains were arranged as a dimer of dimers with a butterfly-like shape (Fig. 1C). The two heterodimers interacted by the C-terminus of nsp16 and as well as the N-terminus of nsp10. We refer to this crystal form as the “large unit cell”.

The overall structure of nsp16-nsp10 in the large unit cell was almost identical to the small unit cell structure, including the bound ligands SAM and Zn^2+^. In order to corroborate the degree of structural identity, the chains of both crystal forms were aligned using the FATCAT server (29). Alignment of nsp16 from the large unit cell (chains A and C) with that from the small unit cell (chain A) showed substantial similarity with a raw root-mean-square deviation (r.m.s.d.) of 0.37 Å and 0.42 Å, respectively. The introduction of a flexibility factor in the alignment showed an optimized r.m.s.d. of 0.38 Å for chain A and 0.77 Å for chain C, demonstrating that the nsp16 structures in these two crystal forms are similar but have flexible regions. One of these flexible regions was a part of the loop formed by the residues Asp^6931^-Phe^6947^, which was disordered from the residues Lys^6933^ to Lys^6939^ in chain C, a likely explanation for the structural differences (movie S1). The nsp10 alignment had a raw r.m.s.d. of 0.23Å for chain B and 0.34 Å for chain D and there were no gaps in the alignment. The optimized r.m.s.d. was 0.28 Å and 0.35 Å, respectively, indicating very low differences between these chains. Thus, both crystallographic forms were almost identical with small discrepancies caused by different conformations in the flexible loops of nsp16.

To determine which stoichiometry existed in solution, we performed analytical size-exclusion chromatography (SEC). In the elution profile, we observed a prominent elution peak at 15 ml that corresponded to a molecular weight (m.w.) of 45 kDa, which is close to the estimated m.w. of the heterodimer (49.8 kDa). We also observed a small peak at ~17.5 ml, containing mostly nsp10 (Fig. 1, D and E). No peak was detected corresponding to ~90 kDa, which would be consistent with four chains in a complex in solution. Thus, the heterodimer is the most soluble and stable form of the 2-*O*-MTase complex, and the dimer of dimers in the large unit cell formed as a result of crystal packing.

### Topology of nsp16 and nsp10 and the heterodimer interface

**Fig. 2.**
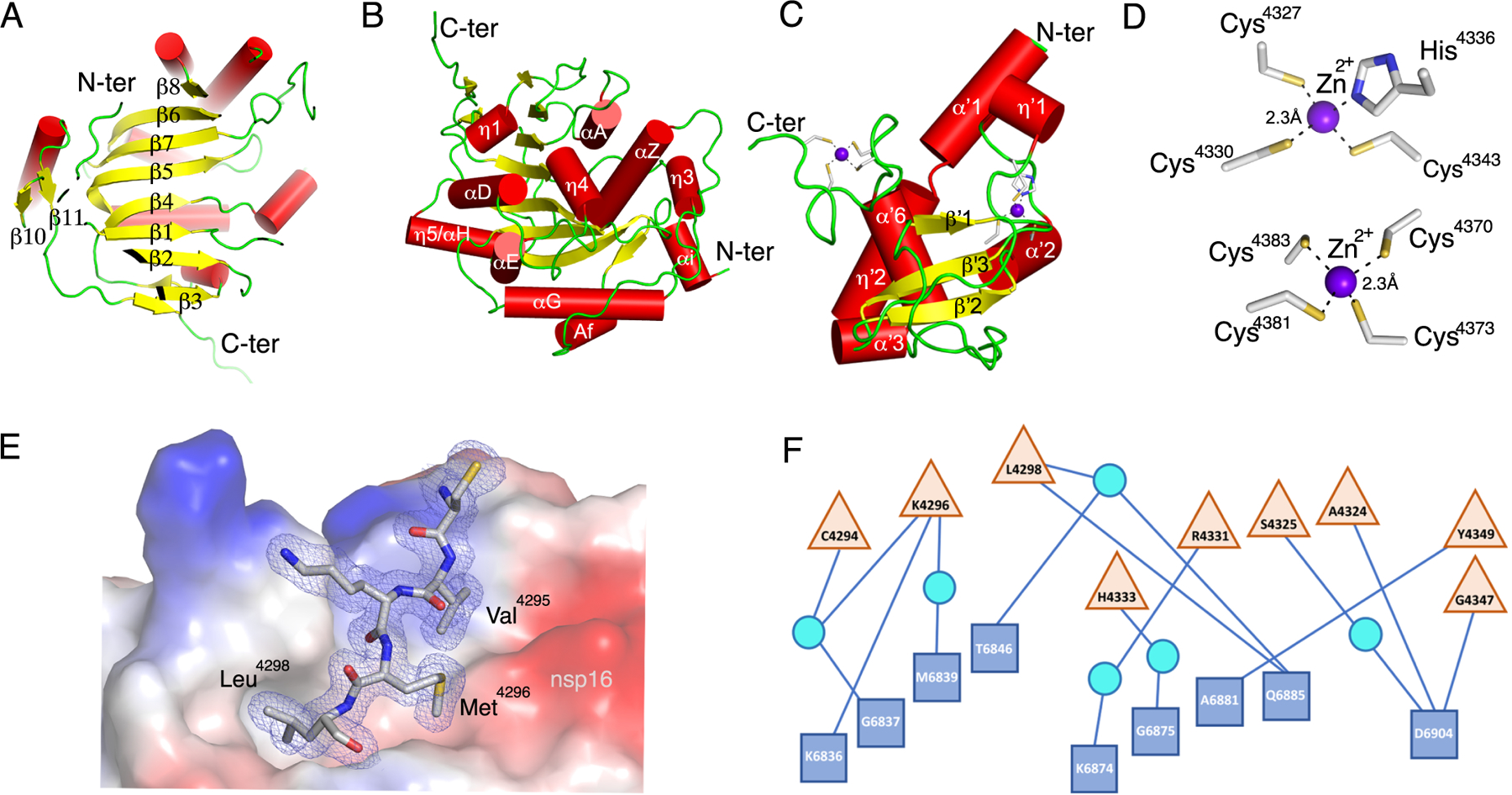
Detailed representation of nsp16, nsp10, and the heterodimer interface. **(A to C)** Cartoon representations of two views of nsp16 featuring the canonical β-sheet (A) and overall secondary structure of nsp16 (B) and nsp10 (C). α-helices are shown as red cylinders, β-strands as yellow arrows, loops as green strands, and zinc ions as purple spheres. **(D)** Close-up view of the two Zn^2+^ binding sites in nsp10. **(E)** Interaction of Cys4294-Leu4298 (sequence CVKML, grey sticks) from nsp10 with the hydrophobic surface of nsp16 (colored by electrostatic potential). Oxygen, red sticks; nitrogen, blue sticks; sulfur, yellow sticks. **(F)** Schematic representation of residues from nsp16 (blue squares) and nsp10 (tan triangles) that interact through hydrogen bonds, represented as lines. Some interactions are mediated by water molecule (cyan circles). For panels A to D, structural representations are based on the structure of the nsp16-nsp10 complex with m^7^GpppA and SAM (PDB code 6WVN). E and F are based on the structure of the nsp16-nsp10 complex with SAM in the small unit crystal form (PDB code 6W4H).

The nsp16 protein consisted of pp1ab residues 6799–7096 plus three additional residues (Ser-Asp-Ala) at the N-terminus derived from the recombinant expression tag after tobacco etch virus (TEV) (20) protease cleavage. The 2′-*O*-MTase catalytic core was comprised of a Rossmann-like β-sheet fold with the canonical 3-2-1-4-5-7-6 arrangement, in which β7 was the only antiparallel strand (Fig. 2A). This β-sheet was sandwiched by eleven α-helices and 20 loops (Fig. 2B).

The nsp10 protein, consisting of pp1a residues 4272–4392, has at its core three β-strands (β‘1, β‘2, β‘3) that form a central anti-parallel β-sheet. At one side of the β-sheet is the large loop that directly interacts with nsp16 and stabilizes the heterodimer complex. At the other side of this β-sheet there are six helices and loops that form two zinc fingers (Fig. 2C). In other coronaviruses, these zinc fingers are involved in non-specific binding of RNA (23, 24). The Zn^2+^-binding site 1 is coordinated by the residues Cys^4327^, Cys^4330^, Cys^4336^, and His^4343^. The Zn^2+^-binding site 2 is coordinated by Cys^4370^, Cys^4373^, Cys^4381^, and Cys^4383^ (Fig. 2D).

The residues forming the nsp16-nsp10 heterodimer interface can be divided into clusters. The clusters for nsp16 are defined as A (residues 6835–6846), B (6874–6889), C (6900–6908) and D (7042–7046). For nsp10 they are defined as clusters I (4293–4300), II (4322–4337), and III (4346–4349) (18, 23). Almost all of the interface contacts between nsp16 and nsp10 are formed by hydrophobic interactions between cluster I (Val^4295^, Met^4297^, Leu4^298^) of nsp10 and cluster A (Pro^6835^, Ile^6838^, Met^6839^, Val^6842^, Ala^6843^), cluster B (Val^6876^, Pro^6878^, Ala^6881^), and cluster D (Leu^7042^, Met^7045^) of nsp16 (Fig. 2E). We observed that the remaining interactions at the interface are mediated by hydrogen bonds, and these hydrophilic interactions consisted of five direct contacts between residues Lys^4296^, Leu^4298^, Ala^4324^, Tyr^4349^, and Gly^4347^ of nsp10 with Lys^6836^, Gln^6875^, Ala^6881^, and Asp^6904^ of nsp16, respectively, plus eight water-mediated interactions (Fig. 2F).

### The binding of SAM, SAH and the inhibitor SFG to the methyl donor binding site

**Fig. 3.**
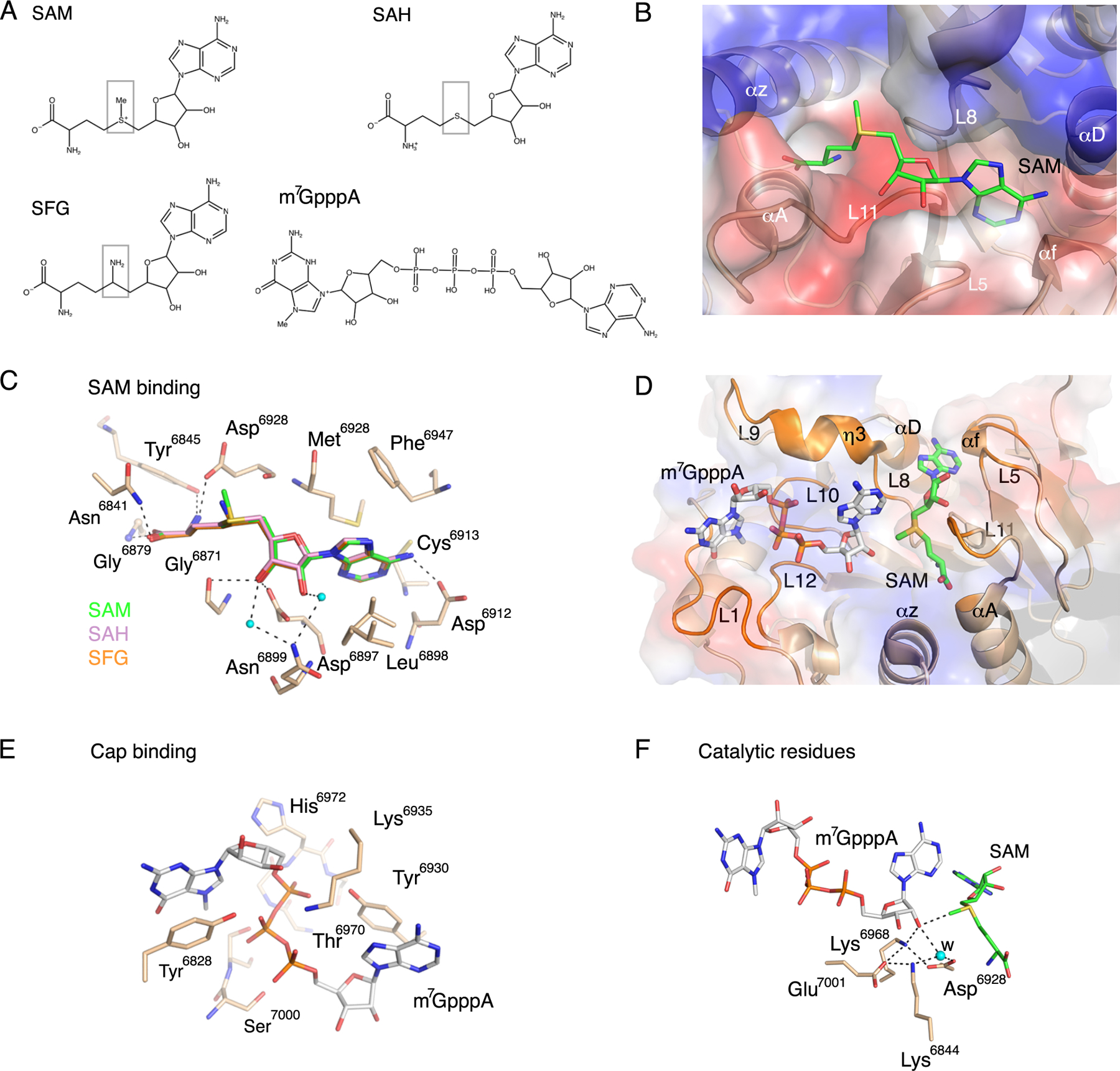
Substrate interactions and catalytic site. **(A)** Chemical structures of the methyl donor SAM; the product after methyl transfer, SAH; the SAH analog and inhibitor, SFG; and the Cap-0 analog, m7GpppA. Boxes highlight differences in the chemical structures of SAH and SFG compared to SAM. **(B)** Cartoon and surface charge representations of the nsp16 SAM binding cleft occupied by SAM (green sticks). **(C)** Close-up view of the overlay of nsp16 structures with the ligands SAM (green, PDB code 6W75), SAH (pink, PDB code 6WJT) and SFG (orange, PDB code 6WKQ). **(D)** Cartoon and surface charge representations of nsp16 (PDB code 6WVN) with the SAM binding cleft occupied by SAM (green sticks) and the Cap binding site occupied by m7GpppA (gray sticks). **(E)**. Detailed view of the residues that coordinate m7GpppA in the Cap binding site as tan sticks. **(F)** Close-up view of the side chains of the catalytic residues showing the orientation of the methyl group in SAM in proximity to the acceptor 2’-OH group in m7GpppA. Dashed lines indicate the interactions between the residues in the active site, and small cyan dots indicate water molecules (w). Red sticks, oxygen; blue sticks, nitrogen; orange sticks, phosphate; yellow sticks, sulfur.

The nsp16 protein catalyzes the transfer of the methyl group from SAM to the Cap-0, generating the reaction products SAH and Cap-1. This reaction can be inhibited by SFG, a 5′-aminoalkyl analog of SAH used as a pan-inhibitor of methyltransferases (Fig. 3A). In order to identify potential structural differences caused by having SAM, SAH, or SFG in the SAM-binding cleft, we also determined the structures of nsp16-nsp10 in complex with SAH (PDB code 6WKQ) and SFG (PDB code 6WJT) at 2.0 and 1.98 Å resolution, respectively. These structures showed that SAM binds to a negatively charged cleft formed by αA, αz, αD, and the loops L5, L8, L11 in nsp16 (Fig. 3B). The adenosine moiety is stabilized by residues Phe^6947^, Asp^6912^, Leu^68^98, Cys^6913^, and Met^6928^. The sugar moiety is stabilized by the residues Gly^6871^ and Asp^6897^ and by two molecules of water that interact with Asn^6899^. The methionine moiety interacts with Asp^6928^, Tyr^6845^, Asn^6841^, and Gly^6871^. Notably, SAH and SFG interact with the same residues as SAM without modifications in the site or the overall structure (Fig. 3C).

### Comparison of nsp16-nsp10 from SARS-CoV-2 and SARS-CoV

At the primary amino acid sequence level, nsp16 from SARS-CoV-2 is 99% identical to Bat-CoV-RaTG13, 94% identical to Bat-SARS-like coronavirus Rs4247(Bat-SL-CoV), and 93% identical to SARS-CoV, but only 66% identical to MERS-CoV (Fig. S1A). The primary amino acid sequence of nsp10 is 100% identical to Bat-CoV-RaTG13, 99% identical to SARS-CoV, and 98% identical to Bat-SL-CoV, but only 59% identical to MERS-CoV (Fig. S1B). Thus, nsp16 and nsp10 are highly conserved in the lineage B betacoronaviruses (Bat-SL-CoV, Bat-CoV-RaTG13 and SARS-CoV), and less conserved with lineage C betacoronaviruses (MERS-CoV). We compared our structures from SARS-CoV-2 with published structures from SARS-CoV (18, 23) to determine if minor differences in sequence impacted the structures of nsp16 or nsp10. The two amino acid differences in nsp10 between SARS-CoV-2 and SARS-CoV are A4276P and K4366R and do not introduce important structural changes (Fig. S1C). Furthermore, in the aligned sequences, the Zn^2+^-coordinating residues in nsp10 are 100% conserved, emphasizing the importance of the zinc fingers across the coronaviruses (Fig. S1B).

Another difference was identified when our nsp16 structure was aligned with the SARS-CoV nsp16 structure published by Chen *et al.* (23), which has three gaps and an optimized r.m.s.d of 1.19 Å. The differences were localized to loop 1 and helix η3 of SARS-CoV-2 (Fig. S1B). The loop 1 sequence that starts at residue 6829 is DSATL in SARS-CoV-2, compared to ENAVI in SARS-CoV. This loop is flexible and is in a “closed” conformation in SARS-CoV compared with a more “open” conformation for SARS-CoV-2. In addition, η3 in SARS-CoV-2 is modeled as a loop in SARS-CoV, which may be due to differences in the primary sequence of PKTKN compared to PRTKH. All other amino acid differences were solvent-exposed (Fig. S1C), and none of them affected the SAM binding site. Further, the nsp16-nsp10 interface residues identified for SARS-CoV-2 (Fig. 2E,F) are 100% identical to this interface in SARS-CoV (Fig. S1A,B), indicating that the interface is conserved between the two viruses.

Despite the sequence divergence, the overall structure of the SARS-CoV-2 2′-*O*-MTase is very similar to structures available for the MERS-CoV 2′-*O*-MTase (27), with no substantial changes in nsp10 (r.m.s.d. = 0.78 Å). The MERS-CoV nsp16 structure is also very conserved, but the loops L1 and L8–L9 are disordered, which causes an increase in the r.m.s.d. of 0.69 Å. One difference between the SARS-CoV-2 and MERS-CoV 2′-*O*-MTases exists at the nsp16-nsp10 interface. Tyr^4349^ in nsp10 contacts Ala^6881^ in nsp16, two residues that are conserved in SARS-CoV-2 and all other betacoronaviruses. However, in MERS-CoV the corresponding residues are Phe and Ser, respectively (Fig. S1A,B). This Phe substitution in MERS-CoV nsp10 promotes the binding of nsp10 to nsp16 and stimulates the MTase activity of nsp16 (30). The conservation of Tyr at this position in SARS-CoV-2 and SARS-CoV could indicate a positive selection against overstimulation of the MTase in the lineage B betacoronaviruses.

### Binding of SAM and m^7^GpppA to the nsp16 catalytic site

In addition to the methyl donor SAM, the nsp16 catalytic reaction requires a Cap-0-mRNA with an adenosine in position 1 of the single-stranded RNA (m^7^GpppA-RNA). The crystal structure of the ternary complex of nsp16-nsp10 with m^7^GpppA and SAM has been determined for the more distantly related lineage C betacoronavirus MERS-CoV (PDB code 5YNM (27)), but not for the more closely related lineage B betacoronavirus SARS-CoV, in which the binding of the mRNA was previously only modeled (23). Herein, we describe structures of the SARS-CoV-2 nsp16-nsp10 heterodimer in complex with m^7^GpppA and either the methyl donor SAM (PDB code 6WVN) or the product SAH (PDB code 6WQ3 and 6WRZ). Overall, the three were almost identical between the SAM- and SAH-bound structures (r.m.s.d. of 0.12 Å) except for sulfates bound into the RNA-binding groove at different positions in one of the structures with SAH. One of structures with cap and SAM (PDB code 6WVN) was selected for more detailed analysis because it was the most complete, with eight additional residues at the N-terminus of nsp10 compared to the SAM-bound structure (PDB code 6W4H) and is at slightly better resolution (Table S2). The N-terminus in this structure forms an α-helix that seems to be in a more open conformation with respect to the previous structure from MERS (r.m.s.d.=0.84 Å) (27).

The cap analog m^7^GpppA bound to the cap high-affinity binding site (HBS), which is a positively charged surface on nsp16 formed by the loops L1, L8, L9, L10, and L12, αD, and the η3 (Fig. 3D). The guanosine ring of m^7^GpppA is stacked with Tyr^6828^. The phosphate groups were mostly stabilized by side chain atoms of Tyr^6828^, Tyr^6930^, Lys^6935^, Thr^6970^, Ser^6999^, and Ser^7000^, and by the main chain atoms of His^6972^ and Ser^7000^ of loops 10 (residues 6970–6975) and 12 (residues 6994–6997) (Fig.3E). The adenosine sugar interacted with side chain atoms of Lys^6844^, Lys^6968^ and with Asp^6928^ through a water molecule. The adenine moiety was stabilized by stacking interaction with the side chain of Tyr^6930^ (Fig. 3F), and it was in close proximity to the SAM binding cleft. These interactions are also found in the structure of MERS-CoV nsp16 in complex with Cap-0 (PDB code 5YNM (27)).

The high quality of the crystal structure of the nsp16-nsp10 complex with m^7^GpppA and SAM bound facilitated detailed analysis of the catalytic site. The protein nsp16 contains the highly conserved residues Lys^6839^, Asp^6928^, Lys^6968^, and Glu^7001^, comprising the canonical catalytic motif (K-D-K-E) conserved among class I MTases (18, 23, 31). These residues are close to the SAM methyl group that is transferred to the 2′-OH on the m^7^GpppA. The amino group of Lys^6968^ is in close interaction with the 2′-OH of Cap-0 and possibly activates this oxygen for the nucleophilic attack of the methyl group in SAM (Fig. 3F). In the structure of nsp16-nsp10 with the m^7^GpppA and SAM, we detected the presence of one molecule of water (Fig. 3F) that might participate in the stabilization of intermediate catalytic states (18, 23, 32). Although the nsp16 MTase reaction was previously characterized as Mg^2+^-dependent, we did not observe this metal bound, and it is likely that Mg^2+^ is involved only in transitory states of catalysis or stability of the protein as previously suggested for dengue virus 2′-*O*-MTase (33).

### The flexibility of the m^7^GpppA site in nsp16

Previous biochemical studies of SARS-CoV nsp16 demonstrated stabilization of the SAM cleft upon nsp10 binding (18, 23) and improved m^7^GpppA-RNA interaction (23), although there is as yet no structure with cap bound to SARS-CoV nsp16 to identify the conformational changes necessary for cap binding. The HBS is surrounded by Loop 1 (residues 6824–6834) and a loop formed by L8–η-3–L9 (residues 6930–6943) in nsp16. These flexible loops, which were not visible in the previously published structure for MERS, are visible in our structures and thus we could analyze the diverse conformations of nsp16-nsp10 complexes.

**Fig. 4.**
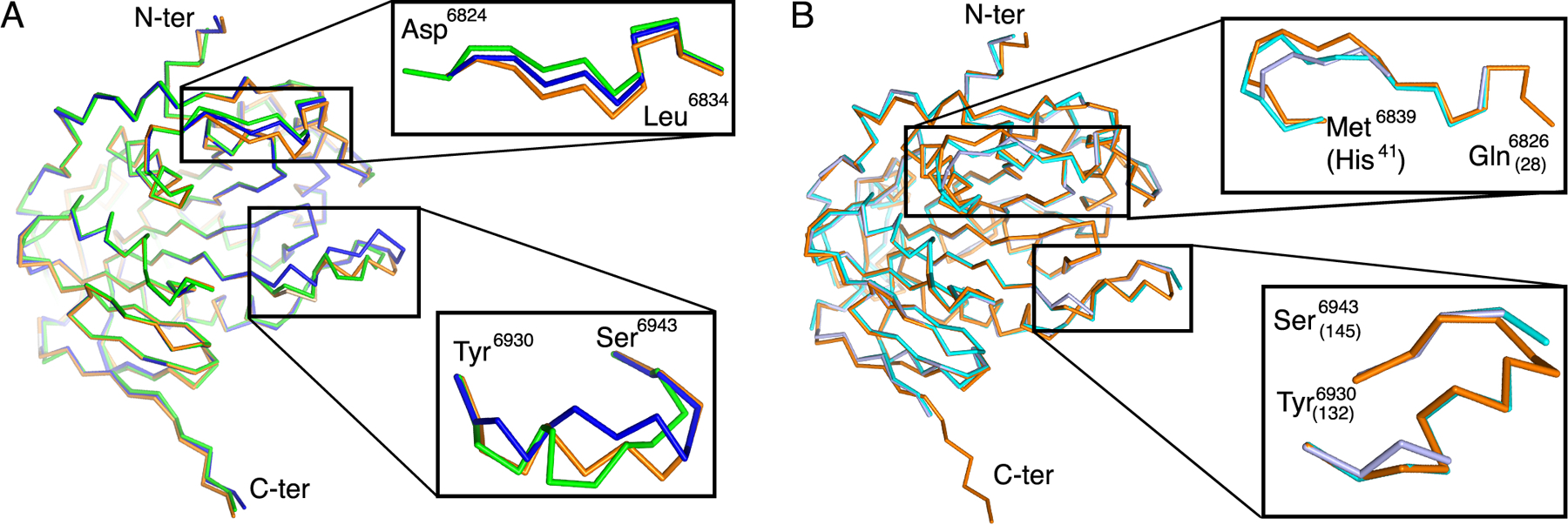
Structural alignment of nsp16 in the presence and absence of m7GpppA. **(A)** Alignment of the C-α chain of nsp16 from SARS-CoV-2 in complex with SAM from the small unit cell (blue, PDB ID 6W4H), in complex with SAM from the large unit cell crystal form (green, PDB code 6W75), and in complex with SAM and m7GpppA (orange, PDB code 6WVN). Two flexible loops are enlarged in insets. **(B)** Alignment of the C-α chain of nsp16 from SARS-CoV-2 in complex with SAM and m7GpppA (orange) with the corresponding region of MERS-CoV nsp16 in complex with SAM alone (light blue, PDB code 5YN6) or in complex with SAM and m7GpppA (cyan, PDB code 5YNM). Numbering of MERS residues is indicated in parentheses.

First, we compared the structures of the heterodimer with only SAM bound from the large unit cell crystal form (Fig. 4A, green) with to that from the small unit cell crystal form (Fig, 4A, blue). There are only minor conformational differences for loop 1 of nsp16, however, the L8–η-3–L9 loop showed a more “open” conformation in the large unit cell and a more “closed” conformation for the small unit cell structure when only SAM was bound. This analysis corroborated that this specific region is flexible and that these loops likely transit from an “open” to a “closed” state in the absence of m^7^GpppA.

In order to evaluate the position of the loops when m^7^GpppA is bound, we analyzed the structural alignment between the high-resolution heterodimer with SAM bound (Fig. 4A, blue) and the heterodimer in complex with m^7^GpppA and SAM (Fig. 4A, orange). This alignment has an r.m.s.d. of 0.55 Å, and we observed that the presence of m^7^GpppA induces a stable open conformation of L8–η-3–L9 (residues 6930–6943), which was found also in the heterodimer structures in complex with m^7^GpppA and SAH (r.m.s.d.=0.12 Å, PDB code 6WQ3 and 6WRZ). The main conformational changes were observed near residues 6936–6939, which were displaced in the open conformation and appeared to mitigate clashes between Lys^6935^ of nsp16 with the ribose and the first phosphate group of the m^7^GpppA and at residue Tyr^6930^ that is rotated to improve stacking interaction with the adenine of the Cap-0 (Fig. S2). These changes indicated that the presence of m^7^GpppA stabilized an open state of the Cap-0 binding site, which could facilitate the release of the product upon methylation.

Furthermore, the C-α chain of nsp16 from SARS-CoV-2 with Cap-0 and SAM bound (Fig. 4B, orange) was aligned with MERS-CoV nsp16 with SAM (Fig. 4B, violet) or with Cap-0 and SAM bound (Fig. 4B, cyan) over 293 residues. In the absence of the Cap-0, the MERS-CoV nsp16 residues corresponding to residues 6934–6940 in SARS-CoV-2 were disordered, confirming that this loop is flexible. In contrast, Loop 1 and residues 6930–6943 from the MERS-CoV m^7^GpppA-nsp16 complex aligns with the open conformation observed in the SARS-CoV-2 m^7^GpppA-nsp16 complex.

### Sulfates align to the RNA binding groove

**Fig. 5.**
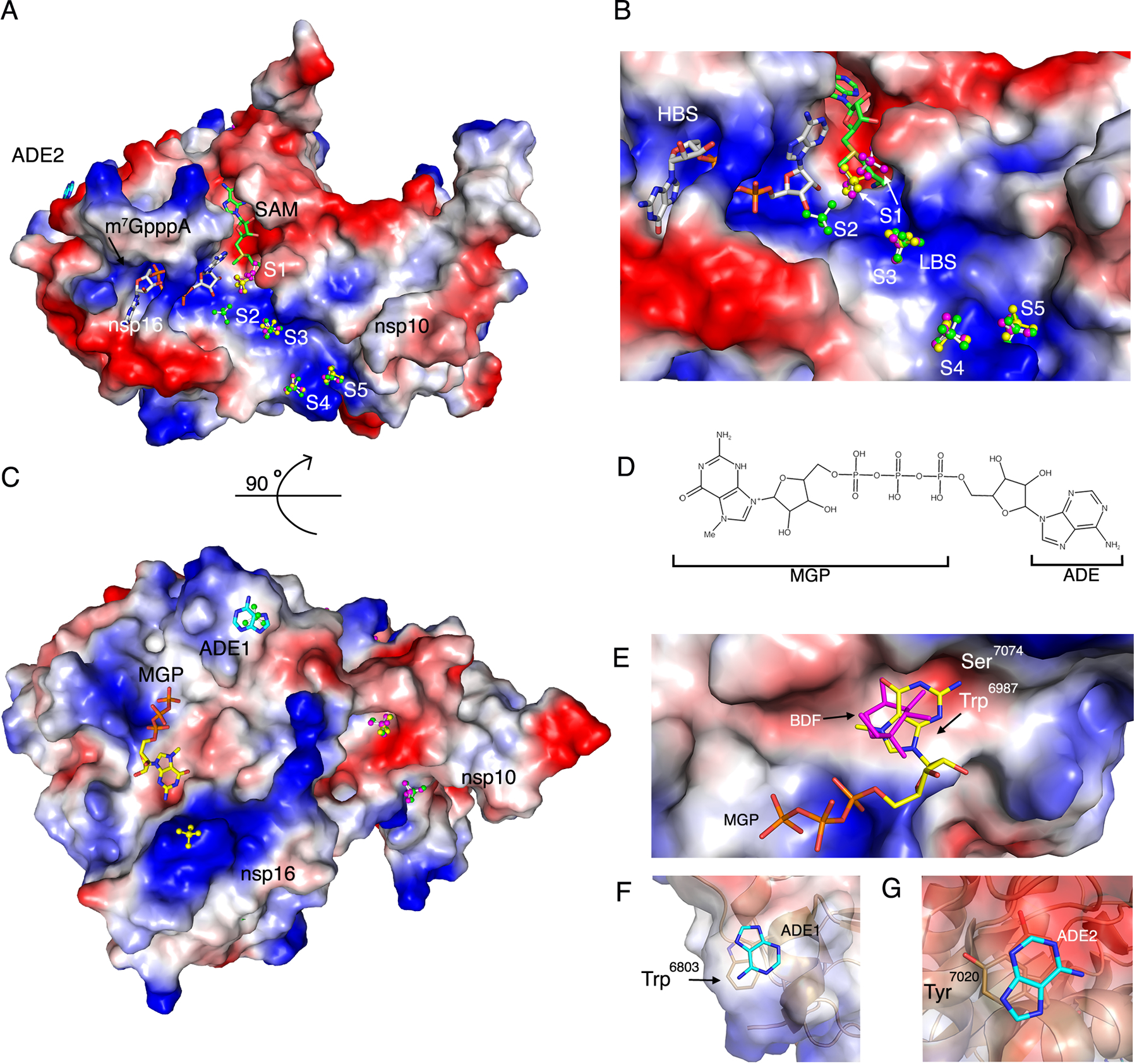
Sulfate and nucleotide binding sites on nsp16. **(A)** Surface charge representation of nsp16-nsp10 bound to SAM and m7GpppA (PDB code 6 WVN) with sulfates in balls and sticks along the nucleotide binding groove numbered from the catalytic core to the nsp10 extension (S1-S5). The sulfates in the overlayed structures are designated by color according with their corresponding PDB code: 6WRZ (green), 6WVN (yellow), and 6WQ3 (pink). m7GpppA is shown as gray sticks and SAM in green sticks. Positive charges are shown in blue and negative charges in red. ADE2, adenine moiety 2. **(B)** Close-up view of (A) showing m7GpppA, SAM and S1–S5 in the high-affinity binding site (HBS) and low-affinity binding site (LBS). **(C)** 90° rotation of the complex showing the secondary binding sites MGP and ADE1 along with additional sulfates. **(D)** Schematic representation of m7GpppA noting the MGP (m7GpppA guanine and phosphate) and ADE (adenine) moieties. **(E)** Surface charge representation of the nsp16 MGP binding site with MGP (yellow sticks) and BDF (pink sticks) from structure 6W4H. **(F and G)** Cartoon and surface charge representation of the adenine moieties (ADE1 and ADE2) bound to nsp16 from structure (PDB code 6WVN). Sticks, carbon; blue, nitrogen; red, oxygen; orange, phosphate; yellow, sulfate.

The 2′-*O*-MTase nsp16-nsp10 possibly binds the viral RNA in the positively charged nucleotide binding groove, also known as the low affinity binding site (LBS). There is, as yet, no structural evidence of the arrangement of this part of the viral RNA in the protein and only predictive models have thus far been published (23, 34, 35). Sulfates are known to bind to proteins in the same positions as nucleic acid backbone phosphates and thus can be used to model nucleic acid binding regions. Because the first small unit cell structure (PDB code 6W4H) was obtained from crystallization conditions with polyethylene glycol (PEG), which did not allow for soaking with substrates, crystals were screened for suitable conditions for soaking with substrates followed by cryoprotection with 2 M lithium sulfate (see Materials and Methods). We speculated that these sulfate molecules could indicate the possible binding sites for the phosphates of the RNA molecule. All of the structures with m^7^GpppA also had molecules of sulfates bound at distinct sites and could be superimposed to analyze the position of sulfates (Fig. 5A–C). Sulfate 1 (S1) was in a position next to the SAM cleft in two alternative conformations, which could indicate the importance of charged molecules near the catalytic site (Fig. 5B). Sulfate 2 (S2) seems to mimic the phosphate group between the first and the second nucleotide in the RNA and is followed by a zig-zag line of other sulfates (S2-S5) along the positively charged LBS from nsp16 and the extension groove of nsp10. The compilation of these results suggested that the nucleotide binding groove might accommodate four to five nucleotides from the viral m^7^GpppA-RNA (Fig. 5B). This experimental and structural evidence reveals the possible position of the viral RNA in the nucleotide-binding groove.

### Identification of three additional m^7^GpppA binding sites in nsp16

The present comprehensive study of the interaction of m^7^GpppA with nsp16-nsp10 resulted in the unexpected finding of nucleotides in non-catalytic sites of the structures (Fig. 5A and 5C). Although, relatively short soaking times were tested to avoid non-specific binding of m^7^GpppA, nucleotides were consistently found at three different positions additional to the active site. One of the sites showed binding of the guanine and phosphate moiety from m^7^GpppA (designated MGP, Fig. 5D) and is located on face of the protein opposite the active site (Fig. 5C). This site was found occupied by a guanine moiety in all three structures with m^7^GpppA. The guanine moiety of m^7^GpppA interacts with the hydrophobic surface formed by Trp^6987^ and with the NH_2_ in the position 2 stabilized by Ser^7074^ in a small negatively charged cavity (Fig. 5E). The adenosine moiety of the m^7^GpppA ligand was disordered. In the structure without the cap (PDB code 6W4H), this same site was occupied by β-D-fructopyranose (BDF) (Fig. 1B and 5E), indicating that this site is not nucleotide-specific.

Another binding site (ADE1) was occupied by an adenine (ADE) moiety that likewise is derived from the m^7^GpppA (Fig. 5C and 5D). The ADE stacked with Trp^6803^ (Fig. 5F). The third binding site (ADE2) is also occupied by an adenine ring stacking with Tyr^7020^ (Fig. 5A and 5G). Although these non-catalytic nucleotide binding events might be crystallization artefacts, they may represent interactions that would mimic the interaction between nsp16 and the ribonucleotides of the capped mRNA.

## Discussion

The SARS-CoV-2 pandemic has yielded an urgent world-wide effort to understand the molecular mechanisms involved in coronavirus transmission, virulence, and replication (1). The ultimate goal is to identify viral proteins that are amenable to drug targeting and epitopes suitable for vaccine development. Although previous studies conducted in the related betacoronaviruses SARS-CoV and MERS-CoV paved the way for drug discovery and vaccine development, no approved treatments were fully developed (36). Thus, in order to ensure an accurate approach for drug discovery, we present a comprehensive study of the first structures of the SARS-CoV-2 2′-*O*-MTase complex that were publicly available to the scientific community. In addition to the structures reported here, similar structures of the nsp16-nsp10 complex have subsequently been determined by other groups; including structures with SAM [PDB codes 6W61 (37), 7BQ7 (38), 7C2I, and 7C2J (39)] and with SFG [PDB code 6YZ1, (34)]. Independently, another structure of nsp16-nsp10 in complex with m^7^GpppA + SAM was also deposited [PDB code 6WKS (35)] but released subsequent to our structure (PDB code 6WQ3).

Previous studies determined that the 2′-*O*-MTases of SARS-CoV and MERS-CoV, which share 93–99% and 59–66% identity to SARS-CoV-2 MTase, respectively, are heterodimers formed by the binding of nsp10 to nsp16 (16, 22). In addition, their structures have provided some insight into nsp10-dependent activation of nps16 MTase catalysis (18, 23, 27). Because variation at the primary sequence level can impact both local and overall structure, ligand binding and structure-based drug design can also be affected by small changes in amino acid sequence. Thus, high resolution structures of the 2′-*O*-MTase from SARS-CoV-2 are necessary to best inform drug discovery for COVID-19.

The present study demonstrated that the binding site for the methyl donor SAM was highly conserved, especially the canonical Gly-X-Gly motif located at the end of the β1 and αA and Phe^6949^, as found in almost all class I MTases (40, 41). SAM analogs have been proposed as antimicrobials targeting MTases of fungi and parasites (42, 43). Here we determined the structure of nsp16-nsp10 bound to the pan-MTase inhibitor SFG and showed that SFG has nearly identical interactions with amino acids side chains as the natural substrate SAM. The high resolution of the structures with bound SAM, SAH, and SFG could facilitate the computational design of small molecules that have higher affinity than SAM for the SAM-binding cleft and have specificity for SARS-CoV-2 nsp16 compared to host methyltransferases. Further, the conservation of the binding cleft residues across the betacoronaviruses (Fig. S1) suggests that an inhibitor designed for SARS-CoV-2 could also be a broader spectrum inhibitor, which could target other coronavirus 2′-*O*-MTases.

The Cap-0 binding site offers another position in nsp16 that could be a target for small molecule inhibition. Although the published structure of nsp16-nsp10 from SARS-CoV was determined with SAM bound, only a computational model of the interaction of SARS-CoV nsp16 with m^7^GpppA-RNA was previously available (23). In order to study the different arrangements in this structure upon Cap binding, crystals were soaked with m^7^GpppA in presence of both SAM and SAH. The resulting structures identified the residues that interact with the Cap-0 and showed that conformational changes in the Cap-0 binding site could occur during catalysis. Of particular concern for the potential development of a more broad-spectrum inhibitor that could target the Cap binding site are two nearby loops that are variable in sequence across the betacoronaviruses, which could have an impact on catalysis (Fig. 5). Our analysis of overlapped structures of nsp16 from the SARS-CoV-2 nsp16-nsp10 complex with the structure of SARS-CoV nsp16 with the Cap binding site unoccupied demonstrated that these amino acid differences do not affect the overall structure of the complex, but rather are highly flexible loops that are then stabilized upon binding of the Cap. Stabilization of these loops may be critical to obtain a high-affinity small molecule inhibitor directed at this site. The high-resolution structure of SARS-CoV-2 with the cap bound should facilitate the design of such a molecule.

A computational model of SARS-CoV 2′-*O*-MTase in complex with RNA was proposed previously using the structure of vaccinia virus MTase (PDB code 1AV6) as the model (23, 34, 35). This model suggested that Asp^75^ in SARS CoV (Asp^6873^ in SARS-CoV-2), confers the selectivity of the Cap binding site for m^7^GpppA over m^7^GpppG due to steric hindrance of the Asp^6873^ residue with the NH_2_ at position 2 of the guanidyl (23). This residue is conserved in SARS-CoV-2. However, in all our structures with Cap bound, the position 2 of the adenylate from m^7^GpppA is 7 Å away from the oxygen of the Asp^6873^, suggesting this residue is unlikely to be involved in the selectivity of the RNA-capped substrate in SARS-CoV-2. Indeed, recent studies show that m^7^GpppG-RNA is 2′-*O*-methylated by nsp16-nsp10 from SARS-CoV-2, but at a lower efficiency than is m^7^GpppA-RNA (35, 44), indicating that this site can accommodate m^7^GpppG.

In addition to the ligand binding sites, we explored the positively charged nucleotide groove, or LBS, which leads from the catalytic core of nsp16 toward nsp10. Several efforts to obtain a short RNA bound into this groove of crystals have thus far been unsuccessful, but several computational models have been recently published and are already available for SARS-CoV (23) and SARS-CoV-2 (23, 34, 35). However, no structural evidence of RNA binding has been reported. Thus, in order to obtain experimental evidence of the possible accommodation of the phosphate groups for four to five ribonucleotides of the mRNA that will directly interact with the nsp16-nsp10 groove, we cryoprotected crystals with lithium sulfate. We speculate that small charged molecules could be designed to prevent binding of mRNA, which could impair the efficiency of the MTase reaction. The potential advantage of targeting a site away from the SAM or Cap binding sites could prevent potential toxicity by avoiding cross-inhibition of human MTases.

To this end, our study revealed previously unknown features in the nsp16-nsp10 structure that could be advantageous for the design of new therapeutics. First, adenine was found to bind at two different sites, stacking with Tyr^7020^ and Trp^6803^ when the crystal was soaked with m^7^GpppA. Further, another possible nucleotide binding site occupied by a guanine moiety was identified on the back of surface of nsp16. This site was also independently identified by another group as occupied by adenosine (35). In addition, we observed BDF in this site when crystals were cryoprotected with sucrose in the absence of m^7^GpppA, suggesting this site is non-specific. Thus, this binding site needs to be further studied in order to determine whether the binding of other molecules might affect the activity of the enzyme, and, if so, whether these newly defined binding sites could be used as potential candidates for developing inhibitors.

A final focus for development of inhibitors against nsp16 is to target the interface with its activator nsp10. Peptides derived from nsp10 have been developed to target SARS-CoV nsp16 in order to impair nsp10 binding to nsp16, resulting in inhibition of nsp16 MTase activity (45). In our analysis, we found that the residues that form the interface between nsp16 and nsp10 are 100% conserved with SARS-CoV. Thus, we predict that small molecules or peptides that target the nsp16-nsp10 interface could also be highly effective inhibitors of nsp16 from SARS-CoV-2 and closely related coronaviruses. An advantage of an inhibitor that instead binds specifically to nsp10 could have even broader implications and potentially also inhibit the N^7^-MTase nsp14, which is also activated by binding to nsp10 (16, 46).

One problem for the development of antiviral compounds against SARS-CoV-2 is the potential impact of emerging mutations in its genome. Newly emerging mutations in SARS-CoV-2 have recently been mapped onto the structures of the proteins they affect (https://coronavirus3d.org) (7). Few mutations have emerged for nsp10, and none of those are predicted to affect the structure of the protein. Some emerging mutations have been identified for nsp16, but likewise none of these are predicted to disrupt the structure. Further, review of these data showed that no mutations of residues at the interface between nsp16 and nsp10 have yet been detected in SARS-CoV-2 viral variants isolated from around the globe (7). Mutations that affect nsp16 activity may be absent from clinical isolates because a reduction or failure in capping could result in early immune detection and accelerate the clearance of the virus. In support of this, recent studies have suggested that stimulation of an interferon response early in SARS-CoV-2 infection could result in less severe disease, particularly in younger individuals (47). In addition, mice infected with Mouse Hepatitis Virus (MHV) showed improved survival when treated with a 29 amino acid peptide, based on the loops of nsp10, that inhibits nsp16 activation. The protection was correlated with higher levels of interferon during early infection, resulting in lower viral titers (45). Altogether, this analysis suggests that immunological studies of the impact of nsp16 inhibition could benefit from the identification of an inhibitor that can be employed for study of the virus in vitro or in animals. Such an inhibitor could also be used during early infection to stimulate immunity, reducing the likelihood for development of severe disease. The structural work found in this study will help with these next stages in our understanding of MTase-mediated modification of viral mRNA and improved treatments for COVID-19.

## Materials and Methods

### Chemicals and synthetic DNA

Common biological chemicals were obtained from MilliporeSigma or ThermoFisher unless otherwise indicated. DNA sequences corresponding to the predicted amino acid sequence for nsp10 and nsp16 from SARS-CoV-2 isolate Wuhan-Hu-1 (NC_045512) were codon optimized for expression in *E. coli* using GenSmartTM Codon Optimization followed by manual editing. The genes were synthesized and cloned into the pMCSG53 vector (48) by Twist Biosciences (South San Francisco, CA). The vector sequences add a TEV cleavable N-terminal 6xHis-tag to expressed proteins. The plasmids were transformed into competent *E. coli* BL21(DE3)(Magic) cells (49).

### Protein expression, purification and analytical SEC

Transformed *E. coli* cells were cultured for expression in Terrific Broth medium (BD Difco) supplemented with 200 μg/ml ampicillin and 50 μg/ml kanamycin incubated at 37°C and 220 rpm. Protein expression was induced at OD600=1.8–2 by an addition of 0.5 mM isopropyl β-d-1-thiogalactopyranoside (Research Products International) and the cultures were further incubated at 25°C, at 200 rpm for 14 h (50). The cells were harvested by centrifugation and resuspended in lysis buffer (50 mM Tris, 0.5 M NaCl, 10% glycerol, 0.1% IGEPAL CA-630) and frozen at -30°C until purification.

Frozen suspensions of cells with expressed nsp10 or nsp16 were thawed and sonicated at 50% amplitude, in 5 s × 10 s cycle for 20 min at 4°C. The lysate was cleared by centrifugation at 18,000 × *g* for 40 min at 4°C, the supernatants were collected, and the protein was purified as previously described with some modifications (51). Each supernatant was loaded into a His-Trap FF (Ni-NTA) column using a GE Healthcare ÅKTA Pure system using loading buffer (10 mM Tris-HCl pH 8.3, 500 mM NaCl, 1 mM Tris(2-carboxyethyl) phosphine (TCEP), 2 mM MgCl_2_ and 5% glycerol). The column was washed with loading buffer, followed by 10 mM Tris-HCl pH 8.3, 500 mM NaCl, 25 mM imidazole, and was eluted with 10 mM Tris pH 8.3, 500 mM NaCl, 1 M imidazole. The protein was loaded onto a Superdex 200 26/600 column and ran with loading buffer, collected and incubated with TEV-protease overnight. The cleaved tag and TEV protease were separated from protein by Ni-NTA-affinity chromatography using loading buffer and Nsp10 was collected in the flow through, whereas nsp16 eluted with 50 mM imidazole. To form the nsp16-nsp10 complex, the pure proteins were mixed at a 1:1 molar ratio at approximately 2 mg/ml in loading buffer and incubated for 1 h, and then dialyzed in crystallization buffer (10 mM Tris-HCl pH 7.5, 150 mM NaCl, MgCl_2_, TCEP and 5% glycerol) for 2 h (26). SAM was added to a final concentration of 2 mM. The complex was concentrated to 5.5–10 mg/ml and set up for crystallization immediately.

In order to confirm that the nsp16-nsp10 complex was formed, we performed analytical SEC using a Superdex 200 10/30 column (GE Healthcare) with 10 mM Tris-HCl, pH 7.5, 150 NaCl, 2 mM MgCl_2_, 1 mM TCEP and 5% glycerol. The standard calibration curve was obtained using combined low molecular weight (LMW) and high molecular weight (HMW) Gel filtration Calibration kits (GE Healthcare). The resulting peaks from the elution of the protein were fractionated in 0.5 ml. Each fraction was collected and 8 μl of sample was denatured with Laemmli buffer (Bio-Rad), then separated using 4–15% gradient SDS-PAGE (Bio-Rad).

### Crystallization, soaking and cryoprotection conditions

The nsp16-nsp10 complex + SAM was set up as 2 μl crystallization drops (1μl protein:1μl reservoir solution) in 96-well (Corning) plates using commercially available Classics II, PEG’s II, AmSO_4_, Anions and ComPAS Suites (Qiagen). Diffraction quality crystals appeared after 5–10 days in 78 conditions, 118 crystals of various complexes were frozen, and 57 data sets were collected. The crystals were soaked, cryoprotected and flash frozen for data collection as follows.

The small unit cell crystal (6W4H) was cryoprotected with 25% of sucrose in the well solution and the large unit cell crystal (6W75) – with 4M sodium formate (Table S1). In order to obtain complexes with SAH and SFG, crystals were transferred into the 10 μl drops with their well solutions supplemented with 5mM of SAH or SFG, soaked for 3 h, cryoprotected with 4M sodium formate or 2M LiSO_4_ and flash frozen. In the attempt to observe the complexes of nsp16-nsp10 with SAM + m^7^GpppA and SAH + m^7^GpppA crystals were transferred into 10 μl drops containing 5 mM SAM or SAH and 0.5 mM of m^7^GpppA in their respective well solutions, soaked for various amount of time from 3 min to 6 hrs and flash frozen. 2 M LiSO_4_ was used as a cryoprotectant in an attempt to observe the binding of sulfates on the places of phosphates from the RNA in the RNA binding groove. Crystals grown in PEG conditions were found not suitable for these soaks due to a phase separation of m^7^GpppA in presence of PEG.

### Data collection and structure determination

Almost 120 crystals were screened, and 57 data sets were collected at the Life Sciences-Collaborative Access Team (LS-CAT) beamlines D, G and F at the Advanced Photon Source (APS) at the Argonne National Laboratory. All the data sets reported here were collected at the beamline F. Images were indexed, integrated and scaled using HKL-3000 (52). Seven structures were chosen to be described in this manuscript (Table S2).

The first structure of nsp16-nsp10 from SARS-CoV-2 in complex with SAM with the small unit cell was determined by Molecular Replacement with Phaser (53) from the CCP4 Suite (54) using the crystal structure of the nsp16-nsp10 heterodimer from SARS-CoV as a search model (PDB ID 3R24, (23)). For all other crystal structures, refined structure from this crystal form was used as a search model. The initial solutions went through several rounds of refinement in REFMAC v. 5.8.0258 (55) and manual model corrections using Coot (56). The water molecules were generated using ARP/wARP (57), SAM, SAH or SFG, Zn^2+^ and ligands were added to the model manually during visual inspection in Coot. Translation–Libration–Screw (TLS) groups were created by the TLSMD server (58) and TLS corrections were applied during the final stages of refinement. MolProbity (59) was used for monitoring the quality of the model during refinement and for the final validation of the structure. A total of seven structures were deposited to the Protein Data Bank (https://www.rcsb.org/) with the assigned PDB codes 6W4H, 6W75, 6WJT, 6WKQ, 6WQ3, 6WVN, and 6WRZ with associated validation reports including electron density maps of all ligands of interest.

### Sequence and structural alignment

The protein sequence of nsp16 and nsp10 from SARS-CoV-2 (YP_009725295.1), Bat-CoV-RaTG13 (QHR63299.1) and Bat-SL-CoV Rs4247 (ATO98179.1) SARS-CoV-1 (ACZ72252.1) and MERS-CoV (YP_009047238.1) were obtained from the NCBI database. The multiple sequence alignment was performed using Clustal-O (https://www.ebi.ac.uk/Tools/msa/clustalo/) and merged with the coordinates of the structure deposited as PDB code 6w4h using ESPript 3.x (60). The PDB coordinates of SARS-CoV nsp16 and nsp10 were analyzed on the FATCAT (29) and PDBFlex servers (61) to perform structural, flexibility and sequence alignment. Structural alignments and structure figures were downloaded from the servers and modeled in PyMOL open source V 2.1 (62). The movie showing the flexibility of nsp16 was generated with the files downloaded from PDBFlex server, frames were captured in PyMOL and exported to editor iMovie editor.

## Supplementary Material

Supplementary Material Fig. S1. Sequence alignments of nsp16 and nsp10 proteins from betacoronaviruses.
Fig. S2. Displacement of the Lys^6935^ and Tyr^6930^ upon m^7^GpppA binding.
Table S1. Crystallization, soaking, and cryoprotection conditions.
Table S2. Crystallographic data.

SM movie Movie S1. The flexibility of the Cap binding site of nsp16.
