# Supplementary material for: High-resolution structures of the SARS-CoV-2 2’-O-methyltransferase reveal strategies for structure-based inhibitor design
Source: Sci Signal. Author manuscript; Available in PMC 2021 Apr 8. (PMC8028745; doi:10.1126/scisignal.abe1202)
Supplement: Supplementary Material — Fig. S1. Sequence alignments of nsp16 and nsp10 proteins from betacoronaviruses. Fig. S2. Displacement of the Lys6935 and Tyr6930 upon m7GpppA binding. Table S1. Crystallization, soaking, and cryoprotection conditions. Table S2. Crystallographic data. [file NIHMS1647468-supplement-Supplementary_Material.docx]

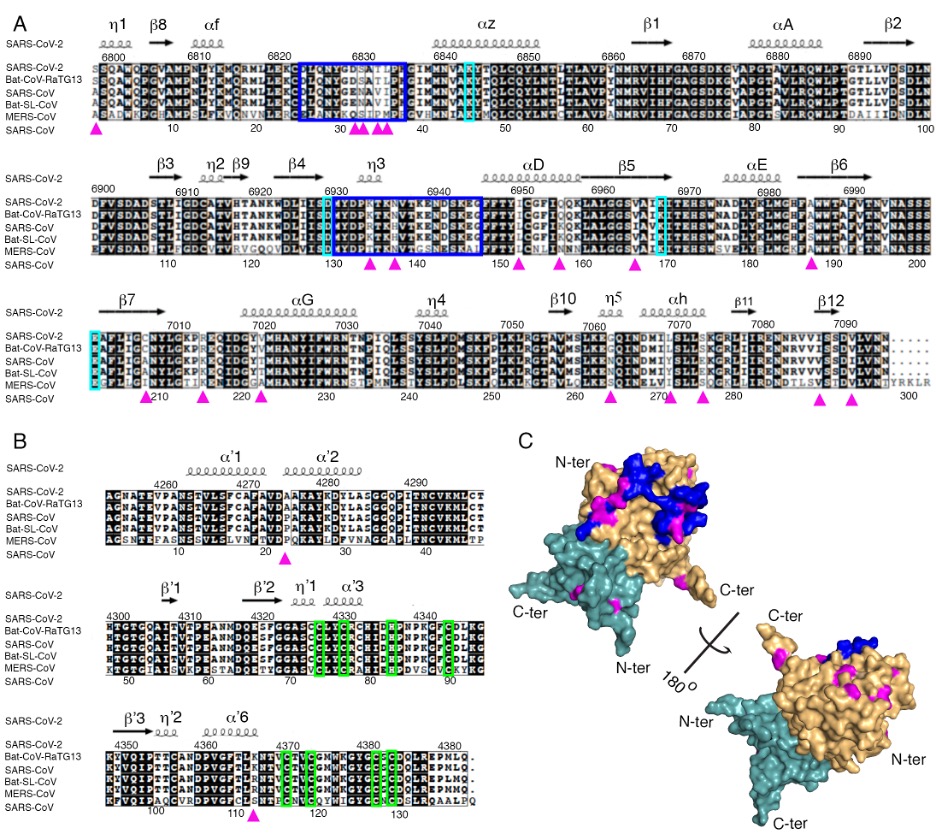
­­­

**Fig. S1. Sequence alignments of nsp16 and nsp10 proteins from betacoronaviruses**. **(A and B)** Multiple sequence alignment of nsp16 (A) and nsp10 (B) from the indicated betacoronaviruses. Black shading indicates 100% amino acid identity across all the betacoronaviruses. Differences between SARS-CoV-2 and SARS-CoV are noted with magenta triangles. Residue numbering according to the pp1ab polyprotein from SARS-CoV-2 is noted on top, and according to the SARS-CoV pp1ab polyprotein from on the bottom of the alignment. In nsp16, the flexible loops involved in Cap and SAM binding are indicated with the dark blue box. The cyan boxes indicate the 100% conserved catalytic KDKE residues. In nsp10, the green boxes indicate the Zn^2+^ binding site. **(C)** Surface projection representation of the structure of nsp16-nsp10 from SARS-CoV-2 with the nsp10 subunit in teal and nsp16 in tan. Single amino acid differences between SARS-CoV-2 and SARS-CoV are mapped in magenta, and the residues comprising the nsp16 flexible loops are mapped in blue.


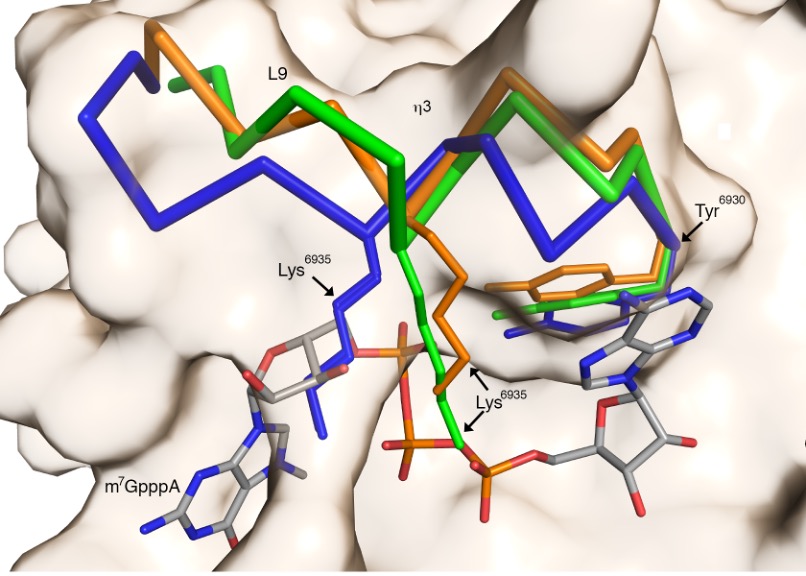


**Fig. S2.** **Displacement of nsp16 Lys^6935^ and Tyr^6930^ upon m^7^GpppA binding.** The flexible loops L8–L9 of nsp16 from the large unit cell with SAM bound (PDB code 6W75, green), from the small unit cell with SAM bound (PDB code 6W4H, blue), and with both SAM and m^7^GpppA bound (PDB code 6WVN, orange) are shown as C-α ribbon. Lys^6935^ and Tyr^6930^ are shown as sticks, and the m^7^GpppA in gray for carbon, red for oxygen, blue for nitrogen, and orange for phosphate. The surface shown in tan corresponds to the small unit cell structure (PDB code 6W4H).

**Table S1.** Crystallization, soaking, and cryoprotection conditions.

| **PDB Accession Code** | **6W4H** | **6WVN** | **6WQ3** | **6WRZ** | **6W75** | **6WKQ** | **6WJT** |
| --- | --- | --- | --- | --- | --- | --- | --- |
| **Protein concentration (mg/ml)** | 5.3 | 5.3 | 5.3 | 5.3 | 9.7 | 5.3 | 5.3 |
| **Screen conditions** | 0.1M HEPES pH 7.5, 0.2M Calcium acetate, 18% (w/v) PEG8000 | 0.1 M HEPES pH 7.5, 0.9M Sodium phosphate, 0.9M Potassium phosphate | 0.1M MES pH 6.5, 0.6M tri-Sodium citrate | 0.1 M HEPES pH 7.5, 0.9M Sodium phosphate, 0.9M Potassium phosphate | 0.01M Tris pH 7.5, 0.4M Potassium/ Sodium tartrate | 0.01 M Tris,  pH 7.5, 0.4M Potassium/ Sodium tartrate | - 1. M Tris,   2. pH 7.5, 0.4M Potassium/ Sodium tartrate |
|  |  |  |  |  |  |  |  |
| **Soaking solution** | ---- | 0.5 mM SAM, 0.5 mM m^7^GpppA | 0.5 mM SAM, 0.5 mM m^7^GpppA | 0.5 mM SAH, 0.5 mM m^7^GpppA | ---- | 0.5 mM SAH | 0.5 mM SFG |
| **Cryoprotectant solution** | 1:1 ratio screen, 50% sucrose | 2M Lithium Sulfate | 2M Lithium Sulfate | 2M Lithium Sulfate | 4M sodium formate | 4M sodium formate | 4M sodium formate |

**Table S2.** Crystallographic data.

| **PDB Accession Code** | **6W4H** | **6WVN** | **6WQ3** | **6WRZ** | **6W75** | **6WKQ** | **6WJT** |
| --- | --- | --- | --- | --- | --- | --- | --- |
| **Data Collection** |  |  |  |  |  |  |  |
| Space group | *P3_1_21* | *P3_1_21* | *P3_1_21* | *P3_1_21* | *P3_2_21* | *P3_2_21* | *P3_2_21* |
| Unit cell parameters (Å; º) | *a = b = 167.7, c = 51.9;*  *α = β = 90.0,*  *γ = 120.0* | *a = b = 169.4, c = 52.1;*  *α = β = 90.0,*  *γ = 120.0* | *a = b = 169.6, c = 52.1;*  *α = β = 90.0,*  *γ = 120.0* | *a = b = 169.14, c = 51.8;*  *α = β = 90.0,*  *γ = 120.0* | *a = b =166.2, c = 98.3;*  *α = β = 90.0,*  *γ = 120.0* | *a = b = 166.2, c = 98.1;*  *α = β = 90.0,*  *γ = 120.0* | *a = b = 166.9 c = 98.1;*  *α = β = 90.0,*  *γ = 120.0* |
| Resolution range (Å) | 30.00 - 1.80 (1.83 - 1.80) | 30.00 - 2.00 (2.03 - 2.00) | 30.00 - 2.10 (2.14 - 2.10) | 30.00 - 2.25 (2.29 - 2.25) | 30.00 - 1.95 (1.98 - 1.95) | 30.00 - 1.98 (2.01 - 1.98) | 30.00 - 2.00 (2.03 - 2.00) |
| No. of reflections | 77,886 (3,873) | 58,029 (2,869) | 50,035 (2,464) | 40,141 (2,001) | 113,483 (5,592) | 108,598 (5,383) | 105,884 (5,270) |
| *R*_merge_ (%) | 6.0 (76.5) | 7.0 (86.7) | 7.5 (81.1) | 7.7 (85.7) | 7.7 (74.2) | 7.1 (78.4) | 10.1 (79.5) |
| Completeness (%) | 100.0 (100.0) | 99.8 (100.0) | 98.8 (98.9) | 99.0 (99.6) | 100.0 (100.0) | 100.0 (100.0) | 100.0 (100.0) |
| 〈*I*/*σ*(*I*)〉 | 29.3 (2.6) | 20.3 (2.1) | 21.1 (2.3) | 23.4 (2.8) | 25.8 (3.2) | 22.2 (2.0) | 16.7 (3.2) |
| Multiplicity | 7.3 (6.8) | 5.1 (5.1) | 7.7 (7.8) | 7.7 (7.3) | 7.6 (7.6) | 5.5 (5.2) | 6.5 (6.6) |
| Wilson *B* factor | 25.4 | 37.4 | 45.4 | 45.1 | 28.4 | 31.7 | 34.2 |
| **Structure Determination** |  |  |  |  |  |  |  |
| MR initial model (PDB ID) | 3R24 | 6W4H | 6W4H | 6W4H | 6W4H | 6W75 | 6W75 |
| **Refinement** |  |  |  |  |  |  |  |
| Resolution range (Å) | 29.76 - 1.80 (1.85 - 1.80) | 29.35 - 2.00 (2.05 - 2.00) | 28.31 - 2.10 (2.15 - 2.10) | 29.91 - 2.25 (2.31 - 2.25) | 29.93 - 1.95 (2.00 - 1.95) | 29.92 - 1.98 (2.03 - 1.98) | 29.79 - 2.00 (2.05 - 2.00) |
| Completeness (%) | 100.0 (100.0) | 99.8 (100.0) | 98.8 (99.1) | 99.0 (99.4) | 99.9 (99.4) | 100.0 (100.0) | 99.8 (98.3) |
| No. of reflections | 73,752 (5,714) | 54,988 (4,221) | 47,173 (3,646) | 38,166 (2,932) | 113,455 (8,247) | 108,429 (7,960) | 100,620 (5,598) |
| *R*_work_/*R*_free_, (%) | 14.9/16.3 (22.9/23.9) | 16.2/17.8 (24.9/25.1) | 16.6/18.6 (23.8/26.3) | 16.2/19.0 (21.2/21.5) | 15.7/17.5 (21.5/23.2) | 16.2/18.0 (25.1/25.4) | 17.1/19/1 (24.5/26.9) |
| Protein chains/atoms | 2/3,201 | 2/3,252 | 2/3,223 | 2/3,214 | 4/6,384 | 4/6,408 | 4/6400 |
| Ligand/Solvent atoms | 65/442 | 197/324 | 148/245 | 166/220 | 125/745 | 107/636 | 97/573 |
| Mean temperature factor (Å^2^) | 31.5 | 46.9 | 53.8 | 53.5 | 33.9 | 38.1 | 40.7 |
| **Coordinate Deviations** |  |  |  |  |  |  |  |
| R.m.s.d. bonds (Å) | 0.004 | 0.004 | 0.005 | 0.005 | 0.005 | 0.004 | 0.006 |
| R.m.s.d. angles (º) | 1.183 | 1.194 | 1.266 | 1.246 | 1.286 | 1.342 | 1.330 |
| **Ramachandran plot** |  |  |  |  |  |  |  |
| Favored (%) | 97.0 | 97.0 | 98.0 | 96.0 | 97.0 | 97.0 | 95.0 |
| Allowed (%) | 3.0 | 3.0 | 2.0 | 4.0 | 3.0 | 3.0 | 5.0 |
| Outside allowed (%) | 0.0 | 0.0 | 0.0 | 0.0 | 0.0 | 0.0 | 0.0 |

**Movie S1. The flexibility of the Cap binding site of nsp16**. The C-α chain of nsp16 is represented as tubing and colored as rainbow from N-terminal (blue) to C-terminal (red). The movie shows the different conformations of the structures solved in this work for nsp16 as a cluster rooted by the Cap-bound PDB code 6WQ3.
